# Differential Network Analysis with Multiply Imputed Lipidomic Data

**DOI:** 10.1371/journal.pone.0121449

**Published:** 2015-03-30

**Authors:** Maiju Kujala, Jaakko Nevalainen, Winfried März, Reijo Laaksonen, Susmita Datta

**Affiliations:** 1 Department of Mathematics and Statistics, University of Turku, Turku, Finland; 2 School of Health Sciences, University of Tampere, Tampere, Finland; 3 Mannheim Institute of Public Health, Medical Faculty Mannheim, University of Heidelberg, Heidelberg, Germany; 4 Synlab Academy, Synlab Services GmbH, Mannheim, Germany; 5 Clinical Institute of Medical and Clinical Laboratory Diagnostics, Medical University of Graz, Graz, Austria; 6 Zora Biosciences, Espoo, Finland; 7 Tampere University Hospital, Tampere, Finland; 8 Department of Bioinformatics and Biostatistics, University of Louisville, Louisville, Kentucky, USA; The University of Hong Kong, HONG KONG

## Abstract

The importance of lipids for cell function and health has been widely recognized, *e.g.*, a disorder in the lipid composition of cells has been related to atherosclerosis caused cardiovascular disease (CVD). Lipidomics analyses are characterized by large yet not a huge number of mutually correlated variables measured and their associations to outcomes are potentially of a complex nature. Differential network analysis provides a formal statistical method capable of inferential analysis to examine differences in network structures of the lipids under two biological conditions. It also guides us to identify potential relationships requiring further biological investigation. We provide a recipe to conduct permutation test on association scores resulted from partial least square regression with multiple imputed lipidomic data from the LUdwigshafen RIsk and Cardiovascular Health (LURIC) study, particularly paying attention to the left-censored missing values typical for a wide range of data sets in life sciences. Left-censored missing values are low-level concentrations that are known to exist somewhere between zero and a lower limit of quantification. To make full use of the LURIC data with the missing values, we utilize state of the art multiple imputation techniques and propose solutions to the challenges that incomplete data sets bring to differential network analysis. The customized network analysis helps us to understand the complexities of the underlying biological processes by identifying lipids and lipid classes that interact with each other, and by recognizing the most important differentially expressed lipids between two subgroups of coronary artery disease (CAD) patients, the patients that had a fatal CVD event and the ones who remained stable during two year follow-up.

## Introduction

Lipids are important for cell function and health, and they have been proposed to be as important for life as proteins and genes. In terms of mass, lipids are the most important constituent of the human brain, and the second most important of all other soft tissues [1]. Lipid metabolism is related as well with several human diseases, such as diabetes, obesity, cancer, and Alzheimer’s disease [2]. Currently, nearly 10 000 different lipids are indexed in the most comprehensive lipid database, LIPID MAPS [3]. Individual lipid species are divided into lipid classes sharing similar structures and biological functions. Proteins and genes are known to have very specific functions. Lipids as structure builders and fat depots are, however, variable, divergent, and versatile. As such, there are no genes coding for lipids. We obtain lipids for example from our diet and they are modified and further modified by gene coded enzymes.

Disorder in the lipid composition of cells has been related to cardiovascular disease (CVD) due to atherosclerosis, the leading cause of death in the United States and most developed Western countries [4]. Early preventive measures depend on accurate identification of patients with an increased risk of CVD. Even though serum total cholesterol and low-density lipoprotein (LDL) cholesterol significantly associate with atherosclerosis and have traditionally been used as a measure of risk, they fail to recognize a substantial proportion of risk patients [5]. Thus, there is a need for more precise understanding of the roles of different lipid species in atherosclerosis beyond LDL cholesterol and HDL (high-density lipoprotein) cholesterol. To meet this goal, we analyze lipidomic data from the LURIC [6] study which provides a well-defined resource for the study of prognostic importance of CVD related common genetic variants and plasma biomarkers. LURIC database contains full GWAS data and over 2000 biochemical variables in addition to full clinical patient characteristics.

The earlier lipidomic findings based partly on the serum samples of the selected LURIC subjects [7] indicate that alterations in sphingolipid (SL) metabolism leading to changes in the fatty acid chain length of ceramides are highly relevant to CV risk. The biochemistry of sphingolipids is currently quite well known, including the elongation processes that leads to molecules of different fatty acid chain lengths. Mathematical models describing the functions and dynamics of the of various metabolic pathways are developing fast [8], but is not easily applied to the sphingolipidome, as the pathways are complex and are not yet completely understood [9]. However, several efforts have already provided better understanding about how sphingolipids are made and function, and also helped interpreting and predicting the outcomes of, for example, genetic mutations [10–12]. On the other hand, the current knowledge covers only the main components of SL metabolism as it has recently been recognized that SL synthesis is very complex, and that each class of SLs contains many different closely related molecular species [13]. It has been so far difficult to map with precision how SL metabolism achieves such level of diversity. It is obvious that there are still central enzymatic steps to be described and metabolites to be identified in order to better understand the behavior of SL metabolism in health and disease. For instance, to identify enzymatic activities responsible for the alterations in SL metabolism linked to cardiovascular diseases will still require significant technological advances. We believe that improved data analysis tools could help to understand the complexities of SL metabolism, which may play a fundamental role not only in CVD pathophysiology, but also in many other diseases including for instance diabetes [14] and central nervous system diseases [15]. SL metabolism is an obvious target for drug discovery and the identification of regulatory steps and pathways for target discovery programs is important.

With the recent growth of mass spectral techniques for lipid profiling and available lipidomic high throughput data, the analysis of lipidomic networks has gained significant interest [16], [17], [18], [19]. Biological association or interaction networks provide information about the essential processes behind different conditions, and help to recognize the important distinguishing lipids, for example for therapeutic purposes. Here, between-lipid interaction (or association) describes the similarity of the concentration levels of two lipids and how they change together. The core of a network analysis is a defined connectivity score that represents the strength of the association or interaction between two particles. At its simplest, the connectivity can be represented with a correlation coefficient. Indeed, previous work on lipidomic networks by Yetukuri et al. [20] is based on between-lipid correlations.

Here we adopt a model based approach to identify important connections between lipids. Fitting a regression model for each lipid as response at a time, with all the other lipids as predictors, enables us to adjust for additional covariates and seek interactions. High throughput data, such as lipidomic data, includes often large number of variables measured in relatively few patients. Common statistical techniques cannot be directly used in such situations, for example, an attempt to fit an ordinary least squares regression model on a data set with more variables than observations would lead to a saturated model. In such cases, one possible solution could be the stepwise regression. Datta [21] showed that in a microarray data a latent variable method, partial least squares (PLS) regression, is a powerful tool for exploring relationships which may translate into biologically meaningful interactions. Later, Pihur, Datta, and Datta [22] proposed a more systematic approach to the PLS-based network construction and showed that PLS based networks outperformed those constructed with simple correlations or partial correlations. Finally Gill, Datta, and Datta ([23]) constructed formal statistical tests on differential connectivities and modular structures based on the PLS-scores. This so called differential network analysis is a method to examine differences in network structures under two biological conditions.

Previous work on differential network analysis was based on a complete case analysis, that is, including only those patients for whom all measurements have been detected. This can lead to a great reduction in the number of patients included, and hence to a serious loss of precision. Due to denoising, left-censored values are a commonplace phenomena for proteomic, metabolomic, and lipidomic data from mass spectrometry platforms. They are low-level concentrations that are considered too imprecise to be reported as a single number with values known to be somewhere between zero and a known lower limit of quantification (LLOQ). Thus, LLOQ is set to filter random noise from the measurements. Finding a proper way to handle the left-censored values is crucial. For example, the LURIC data set used in this analysis does not include any patients with fully detected lipid profiles and majority of the missing lipid concentrations are caused by left-censoring. If patients with left censored values are systematically removed from the analysis—as they would in the complete case analysis—the analysis can be severely biased. Thus, exclusion of the left-censored values produces an upward bias in subsequent measures of location, such as means and medians. Commonly used methods to deal with values below quantification limits are to substitute a fraction of the quantification limit or zero for each non-detect, or single imputation. It is well known that even when there is no systematic pattern of missing values, a complete case analysis accompanied by substitution methods or single imputation is typically biased and the inference invalid. Only the multiple imputation (MI) method, where each missing value is imputed with a set of plausible values, incorporates the uncertainty among imputations into the final inferential procedures.

To make full use of the observed LURIC data with the informative missingness caused by left-censoring, we utilize state of the art MI techniques and propose solutions to the challenges that incomplete data sets and their imputation bring to differential network analysis. The analysis is adjusted for additional covariates, such as age, body mass index, use of statins and smoking status of the patients. This allows us to maximize the use of all relevant information in the data. The ultimate aim is to compare differential network connectivities and modular structures of two subgroups of patients from LURIC data, cases and controls, and identify lipids that are related to increased risk of CVD related death.

In the Materials and methods section, we describe the study design, data collection, and the missing value patterns. We propose particular missing data imputation methodology as well as review the methods for construction of the connectivity scores and corresponding networks along with the hypothesis tests to investigate the differences in the network topology between two networks. Results section provides the implementation of the differential network analysis for the multiple imputed LURIC lipidomics data and we conclude the paper with a discussion on the methods and results.

## Materials and Methods

### Ethics statement

The LURIC study was approved by the ethics review committee at the “Landesärztekammer Rheinland-Pfalz”.

### Study design

The LURIC study is an ongoing prospective study enrolling currently more than 3000 patients with German ancestry [6]. Patients were recruited between years 1997 and 2002 after arriving to one of the research hospitals due to symptoms referring to a CVD. After obtaining a written consent, baseline examination was performed including an individual and family history questionnaire and extensive sampling of fasted venous blood. The coronary artery status was evaluated by angiography.

Lipidomic profiles were measured from a retrospectively defined subgroup of *n* = 445 males, with 258 cases and 187 controls. Detailed description of this lipidomic study, lipid extraction and the mass spectrometry analyses are given in [7]. The main interest lies in comparing the interrelationship of the concentration levels among various lipids between the patients that had a CVD event leading to death during the first three years follow-up (cases) and the ones who survived at least three years (controls). Cardiovascular deaths were defined as sudden cardiac death, fatal myocardial infarction, death due to congestive heart failure, death immediately after intervention to treat CAD, fatal stroke, and other causes of deaths due to cardiac disease. Frequency matching was done to ensure that the case and control groups had the same distributions over strata defined by age, body mass index, statin use and smoking. The number of controls remained smaller than number of cases due to the exclusion of numerous stable diabetic patients having pre-study events indicating plaque vulnerability.

Data access requests about LURIC data [6] should be addressed to Prof. Dr. Winfried März (winfried.maerz@synlab.com) and requests about lipidomic data [7] to Dr. Reini Hurme (reini.hurme@zora.fi).

### Data acquisition

Lipids were extracted from an aliquot of serum. Known amounts of internal standards were added to the samples before extraction. Quantification of lipid concentrations in plasma was done by using mass spectrometry. For each platform containing a set of samples, a stringent cut-off was applied for separating background noise from actual lipid peaks. This cut-off value is called a lower limit of quantification (LLOQ). Acquired mass spectrometry data were processed using bioinformatic tools that covert masses and counts of detected peaks into corresponding lipid names, and by using using the internal standards, transform abundances of molecule masses into concentrations. The concentrations of molecular lipids are presented as *μ*M for serum.

Quality control samples were utilized to monitor the overall quality of the lipid extraction and mass spectrometry analyses by removing technical outliers and lipid species that were detected below the lipid class based LLOQ.

### Missing values and multiple imputation by chained equations

In general, we observe a vector of binary responses **y** = (*y*_1_, …, *y*_*n*_)′ indicating whether the patient was a case or a control, and the log-transformed lipid concentrations **X** = (**x**_1_, …, **x**_*n*_) of the *n* patients. Let *x*_*ij*_ be the (log-transformed) concentration of the lipid *j* for patient *i* (*i* = 1, …, *n*; *j* = 1, …, *p*). Typically, a substantial number of *x*_*ij*_ are not detected. Let **x** ^obs^ and **x** ^mis^ denote the observed and the missing elements in **x**, respectively.

In this context, there are two types of missingness. First, in the presence of an assigned LLOQ, denoted by (say) *l*_*j*_, we have values below *l*_*j*_ that are left-censored, also known as non-detects. As LLOQ is set for each platform, it can vary between different lipid species. Another type of missing values yields due to the elimination of observations not fulfilling the quality control standards. It is reasonable to regard these to be missing completely at random. These two types of missing values and their characteristics are taken into account in the imputation algorithm by imputing them in two different ways, as described in the next section.

MI is a statistical technique for handling missing data and its theoretical foundation is well established. MI is widely used with various “omics”-data sets [24–28]. The key idea is to use the conditional distribution of the observed data to generate a set of plausible imputations for the missing data. In practice, the draws are based on an appropriate posterior distribution [29]. Imputations are repeated *M* times, creating multiple data sets which are analysed individually as if they were complete. Thus, we obtain a set of parameter estimates. Finally, the results are combined across all multiply imputed data sets by averaging them, and the standard errors of the estimates are computed as a combination of within-imputation and between-imputation variances, by so-called Rubin’s rules [29]. These rules incorporate the imputation related uncertainty into the analysis.

From now on, we will omit the index *i* for the ease of notation. The construction of an appropriate imputation distribution is critical. Accordingly, an appropriate imputation model needs to be specified. In terms of the general notation, this is given by

$$\begin{array}{c} f(x^{\text{mis}}|x^{\text{obs}},y,\theta ). \end{array}$$

Here, ***θ*** represents a vector of the regression coefficient parameters consisting of the intercept term and the slope parameters for the other lipids, case/control status and the clinical covariates. Typically, this will be a multivariate regression model specifying the dependence of the conditional distribution of the missing data on the observed data. It is convenient to construct this joint distribution indirectly through a set of univariate conditional regressions, once for each incomplete variable. The choice of the model is flexible depending on the type of the variable to be imputed, e.g. linear regression for continuous variables, and logistic regression for binary variables. This procedure is known as sequential regression imputation strategy, multiple imputation by chained equations (MICE), also known as fully conditional specification [30], [31].

As an initial step, a simple imputation, such as substituting missing values with mean, is performed. Next, one variable at a time is set as a dependent variable, and in that variable, the initially missing values are set back to missing. Then the observed values of the dependent variable are regressed on all the other variables in the imputation model (in our case, other lipids, case/control status of the patient and the clinical covariates). Finally, the missing values in the dependent variable are replaced with draws from predictive distribution given by the regression model. The whole cycle going through all the variables including missing values, with the imputations being updated at each round, is repeated until approximate convergence [30], [32].

As the left censored missing values can be distinguished from the values that are missing completely at random, and the LLOQ *l*_*j*_ is registered, it is essential to incorporate this knowledge into the imputation model. In practice, this brings one additional condition to the imputation algorithm. The imputation is carried out using an acceptance-rejection sampling principle: for left-censored values, draws from the conditional distribution are accepted only if they fall below the LLOQ. If a candidate value does not meet this condition, it is rejected and a new candidate is drawn sufficiently many times until acceptance. In other words, imputations for left censored values are sampled from the left tail of the appropriate conditional distribution. For the values missing completely at random, all draws are accepted.

The multiple imputations were performed with R-package *mice* [33]. It is generally believed that it is safer to overfit an imputation model (include too many variables in the model) than to underfit (omit an important variable) [34], [35]. For this reason, in addition to all detected lipids, information about the case/control status of the patients, age, body mass index, smoking, number of myocardial infarctions, LDL and HDL cholesterol values, total cholesterol, triglycerides, apolipoprotein A-I, C-reactive protein, apolipoprotein B, statin use, lipid lowering therapy, non-statin lipid-lowering treatment, and type II diabetes mellitus was included in the fully conditional specification. The LLOQ is assumed to be known so it does not need to be estimated.

### Stacking the multiple imputed data sets

The differential network analysis can be challenging in the presence of missing values. Although MI solves the missing data problem, how to combine the results from *M* individually analysed MI data sets remains unclear, as different imputations may result in different networks. For the purposes of differential network analysis, we applied a stacking-method proposed by Wood et al. [36]. Instead of running *M* individual analyses, we analyse one large data set with *Mn* rows, resulting from stacking the *M* multiple imputed data sets. This results in each patient being repeated *M* times in the stacked data set.

For the purposes of the network analysis, the stacked data set is centered for mean zero and scaled for unit variance. Stacking does not affect the sample mean, but decreases the sample variance. For centered data, the standard deviation is

$$\begin{array}{c} s^{2}=\frac{1}{n-1}\sum_{i=1}^{n}x_{ij}^{2}. \end{array}$$

If we stack the observations *M* times, the sample standard deviation of the same variable from the stacked data is

$$\begin{array}{c} s_{\mathrm{stack}}^{2}=\frac{M}{M\cdot n-1}\sum_{i=1}^{n}x_{i}^{2}\;=\frac{n-1}{n-\frac{1}{M}}\;s^{2}. \end{array}$$

Consequently, the sample variance obtained from the stacked data can be corrected by multiplying it by $\left(n-\frac{1}{M})/(n-1\right)$.

### Reconstructing a lipidomic association network using partial least squares based connectivity scores

The core of the network analysis is a connectivity score, ${\hat{s}}_{jk}$ between the lipids *j* and *k*, which represents the strength of the association or interaction between two particles. As proposed by Pihur, Datta, and Datta [22], PLS based connectivity scores are achieved by fitting *p* PLS models such that each lipid at a time is predicted with the remaining *p* − 1 lipids. We also adjusted our models for additional covariates including age, body mass index, use of statins and smoking. Different steps of PLS include first computing user selectable number *v* < *N* orthogonal latent factors $t_{j}^{\left(\ell \right)}$ from the data, and then fitting a linear model

$$\begin{array}{c} x_{j}=\sum_{\ell =1}^{v}\beta_{{}_{\ell }}t_{j}^{\left(\ell \right)}+\epsilon . \end{array}$$

We have used *v* = 3 latent variables throughout the analyses. The latent factors $t_{j}^{\left(\ell \right)}$ are linear combinations of lipids *x*_1_, …, *x*_*j*−1_, *x*_*j*+1_, …, *x*_*p*_ with PLS regression coefficients $c_{jk}^{\left(\ell \right)}$ and are sequentially constructed. The connectivity score is finally computed in a symmetrized form revising the roles of lipids *j* and *k* as

$$\begin{array}{c} {\hat{s}}_{jk}=\frac{\sum_{\ell =1}^{v}{\hat{\beta }}_{j\ell }c_{jk}^{\left(\ell \right)}+\sum_{\ell =1}^{v}{\hat{\beta }}_{k\ell }c_{kj}^{\left(\ell \right)}}{2}. \end{array}$$

The regression coefficients relating to the additional covariates are not used in computing the connectivity score.

### Modules of lipids

Biological networks have often a modular structure where lipids belonging to different clusters have a weak or no connection between them, while within a cluster lipids are connected by short paths with strong connections. In an unsupervised study, one goal of the network analysis is to identify such modular structures. Modular structures are mainly examined by visual means, but also a mathematical definition of a module is provided by Gill, Datta, and Datta [23]. Let *m* be the minimum module size parameter, and *ɛ* a certain threshold criteria of the connectivity scores. If the connectivity score between two lipids is above *ɛ*, lipids are included in the network. A collection of lipids is called a module if at least *m* of them are connected by a path of lipids such that the connectivity score between all pairs of lipids on that path is at least *ɛ*. In addition, such a set has to be a maximal collection so that all the connectivity scores between lipids within the module are at least *ɛ* and outside the module are smaller than *ɛ*.

### Differential network analysis

#### Testing for different modular structures in two networks

Building on the works of Gill, Datta, and Datta [23], we provide a modified permutation test on association scores resulted from partial least square regression on stacked multiple imputed lipidomic data.

Let us assume that the two networks have been constructed separately for case and control samples using association scores based on PLS regression. Given the parameters *m* and *ɛ* one can identify the modular structures of the two networks. Let {ℱ_*k*1_, …, ℱ_*kR*_*k*__} be a set of all the distinct modules *r* of size at least *m* and with connectivity *ɛ* in network *k*, for *k* = 1,2. Then *R*_1_ is the number of modules for case network and *R*_2_ the number of modules for control network. Denote ℒ_0_ the collection of all lipids that are present in some module in both networks, ${ℒ}_{0}=\underset{k}{\cap }\underset{r}{\cup }{ℱ}_{kr}$.

Let ℱ_*kr*(*j*)_, for *k* = 1,2, be the module in network *k* that contains lipid *j*, *j* ∈ ℒ_0_. The test statistic

$$\begin{array}{c} 𝒩=1-\frac{1}{|{ℒ}_{0}|}\sum_{j\in {ℒ}_{0}}\frac{|{ℱ}_{1r(j)}⋂{ℱ}_{2r(j)}|}{|{ℱ}_{1r(j)}⋃{ℱ}_{2r(j)}|}, \end{array}$$

captures the differences between two modular structures in the two networks. ∣ℒ_0_∣ gives the number of lipids that belong to some modules in both networks. Let us assume that a given lipid *j* belongs to a module in network 1 which contains lipids {*j*_1_, *j*_2_, *j*_3_} and in network 2 to a module consisting of lipids {*j*_1_, *j*_2_, *j*_4_, *j*_5_}. Then the numerator of the sum in the test statistic 𝒩 is 2 and the denominator 5. Thus, the test statistic gets values between 0 and 1 where zero indicates identical modular structures. An empty sum is to be interpreted as 0.

When implementing the MI method on the data to be analysed, the p-value for the overall modular structure test is obtained using a following permutation scheme.

(i) Multiple impute the original data set of size *n* by using chained equations. Compute and save the test statistic 𝒩 for the centered and scaled multiple imputed stacked data.
(ii) Permute the group statuses (case/control) of the patients of the original data set of size *n*. Permutation can be executed for example by first sorting the data so that *n*_1_ rows are the real cases and last *n*_2_ rows are the real controls. Then we permute the order of the rows and subsequent *n*_1_ first rows are the new cases and *n*_2_ last rows are the new controls.
(iii) Multiple impute the permuted data set of size *n* *M* times and stack the resulting *M* data sets into a one large data set of size *Mn*.
(iv) For each permutation *π*, compute and save the test statistic
  $$\begin{array}{c} 𝒩\left(\pi \right)=1-\frac{1}{|{ℒ}_{0}\left(\pi \right)|}\underset{j\in {ℒ}_{0}\left(\pi \right)}{\sum }\frac{|{ℱ}_{1r\left(j\right)}\left(\pi \right)\cap^{}{ℱ}_{2r\left(j\right)}\left(\pi \right)|}{|{ℱ}_{1r\left(j\right)}\left(\pi \right)\cup^{}{ℱ}_{2r\left(j\right)}\left(\pi \right)|}. \end{array}$$
(v) Repeat steps (ii)–(iv) *P* times.
(vi) Test the null hypothesis H_0_ : 𝒩 = 0, meaning that the modular structures of the two networks are indentical, by computing the p-value $p(𝒩)=\frac{1}{P}\sum_{\pi }I(𝒩(\pi )\geq 𝒩),$ where the sum is taken over all *P* permutations *π*.

All network analyses were performed utilizing the functions of the *dna* R-package and combining them with our own functions for different imputation and permutation schemes. The *dna*-package can be installed from CRAN. Tests with the additional covariates were fitted with an updated version of the *dna*-package, that is not yet publicly available.

#### Testing for differential connectivity of a single lipid

The differential connectivity of a single lipid *j* in two networks can be measured by using a mean absolute distance statistic by Gill, Datta, and Datta [23],

$$\begin{array}{c} d\left(j\right)=\frac{1}{p-1}\sum_{j\prime \in ℒ,j\prime \neq j}|{\hat{s}}_{jj\prime }^{1}-{\hat{s}}_{jj\prime }^{2}|, \end{array}$$

where the sum is taken over all the remaining lipids in a network, and where ${\hat{s}}_{jj\prime }^{k}$ is the connectivity score between lipid pair (*j*, *j*′) in networks *k* = 1,2. The permutation-imputation-computation of the test statistic -scheme is similar as described above for the test of different modular structures in two networks. It is worth noting, that the test statistics and related p-values for all the lipids can be computed simultaneously using the same set of permutations.

For the purposes of reference, we also performed a marginal analysis separately for fully observed and imputed lipids. Lipids including imputed values were analysed by fitting identical ANCOVA-models to five multiple imputed data sets and combining the results using Rubin’s rules [29], [37]. For fully observed lipids, a single ANCOVA-model was fitted. Each of the lipids was explained with the case/control status, age, body mass index, statin use, and smoking.

## Results

Partial lipidomic profiles of 445 CAD patients were quantified and a total of 237 lipids was detected. Of those, 86 lipids were detected in at least 60% of the patients and used throughout the analyses.

**Fig 1:**
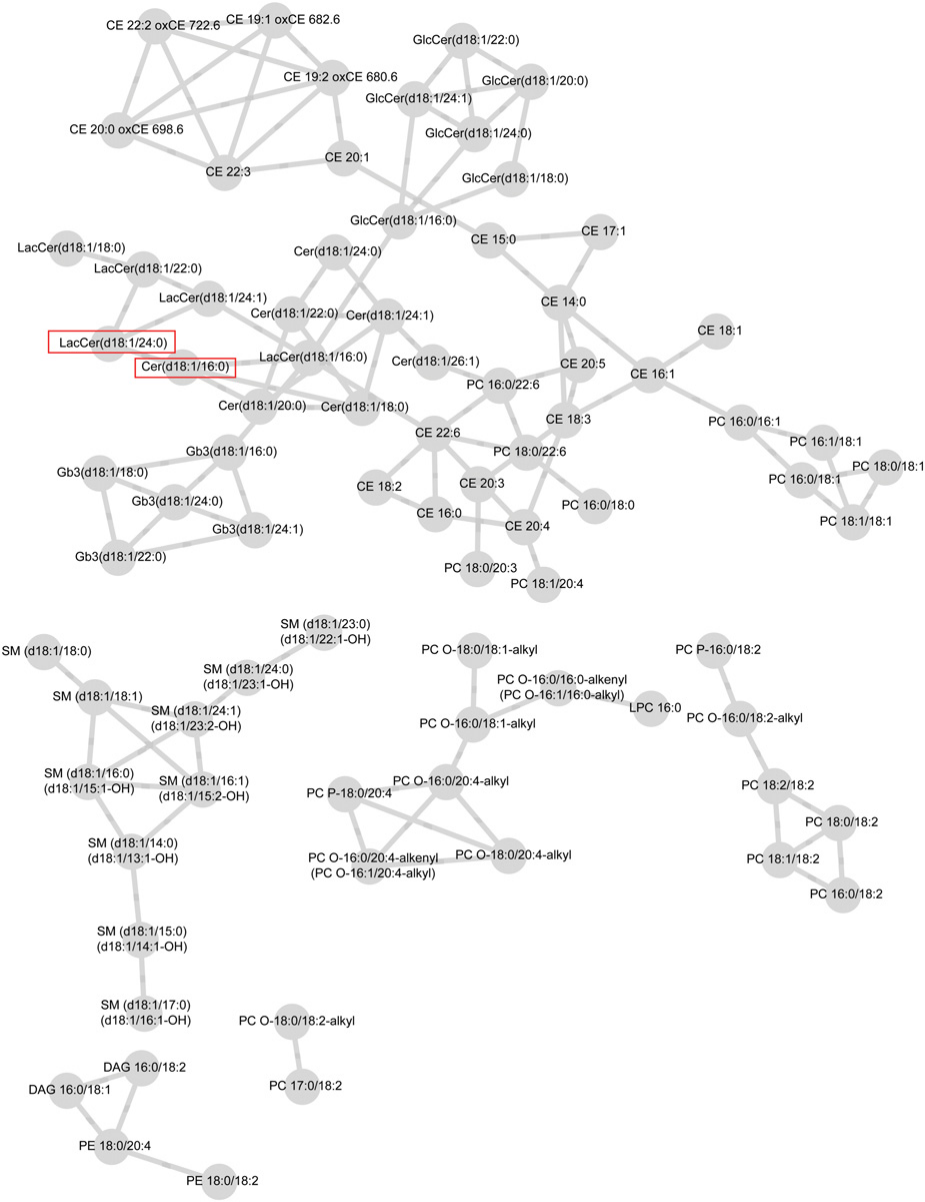
Lipid network for case-group with parameter values ε = 0.4 and minimum modular size = 3. Significant lipids from the test for differential connectivity of a single lipid are circulated. A module consisting of lipids belonging to the same lipid class is highlighted with rectangles as well.

**Fig 2:**
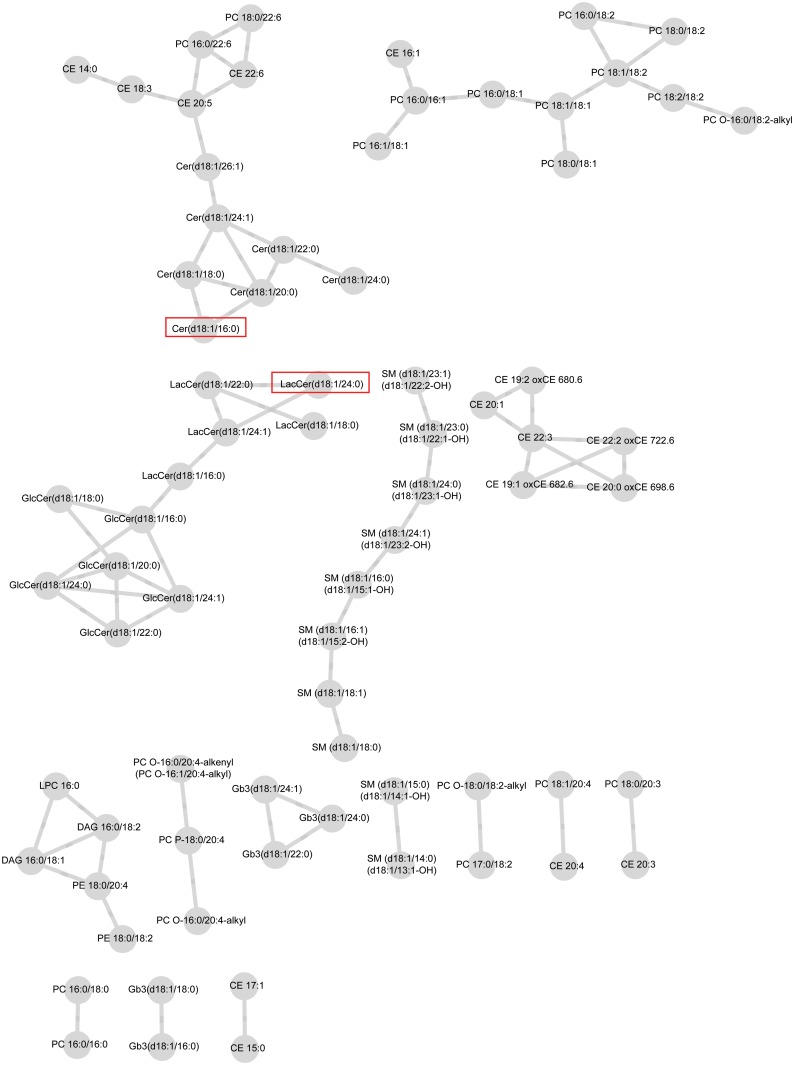
Lipid network for control-group with parameter values ε = 0.4 and minimum modular size = 3. Significant lipids from the test for differential connectivity of a single lipid are circulated. Modules consisting of lipids belonging to the same lipid class are highlighted with a rectangle as well.

Connectivities were rescaled so that the largest score for each lipid was one in magnitude. For both case and control groups, networks including the connectivities above the selected threshold, *ɛ* = 0.4, are visualized in Fig. 1 and Fig. 2. These figures are obtained from the Cytoscape software [38].

The network of case group consists of six modules where as the network for control group has 15 modules. The control group network contains 74 lipids and 78 edges compared to 81 lipids and 118 edges on the case network suggesting, perhaps, higher number of strong connectivities in the case network. In both networks, some of the modules are formed solely by lipid species belonging to the same lipid classes which is natural, as they tend to correlate with each other and have similar biological functions. For example, in the case network (Fig. 1), one module consists only of sphingomyelins (SM). In the control network (Fig. 2), part of the SM class, part of the cholesteryl esters (CE), and part of the globotriaosyl ceramides (Gb3) form their own modules. These modules are highlighted in Fig. 1 and 2. We observed associations of CE and phosphatidylcholines (PC) both in the case and the control groups. In the case network, ceramides (Cer), lactosylceramides (LacCer), and glucosylceramides (GlcCer) were closely associated with each other and formed one large module with CE and PC classes. In the control network, we found associations between PC, CE, and Cer classes while LacCer and GlcCer lipids isolated in their own module.

Based on the test of differential network modular structures with minimum module size *m* = 3 and connectivity threshold *ɛ* = 0.4, case and control groups did not differ significantly on 0.05 level (p = 0.236). However, according to our previous experience it is very difficult to show significant difference between overall modular structures between two groups. For the sake of completeness, we performed the differential network analysis for the modules with different choices of *ɛ*. The results are presented in Table 1. For multiple imputed data, threshold parameters *ɛ* = 0.2 and *ɛ* = 0.25 led to the case and control networks consisting only of one module. Thus, network structures were identical between cases and controls and the test statistic 𝒩 = 0. On the contrary, *ɛ* = 0.65 retained only one module consisting of three lipids in the case network and no modules at all in the control network. In that case, the two networks are fully different and the test statistic 𝒩 = 1. For higher values, *ɛ* > 0.65, networks do not contain any modules. For any reasonable choice of *ɛ* (0.2 − 0.8), the differences in modular structures remain non-significant.

**Table 1:** Test for differential modular structure in the case and control networks for MI LURIC data. For comparison, the same statistics and p-values are given for complete case (CC) data with subgroup of lipids from which 90% of the values were detected.

*ɛ*	𝒩	p-value	𝒩_*CC*_	p-value
0.20	0.00	1.000	0.04	0.530
0.25	0.00	1.000	0.11	0.620
0.30	0.02	0.582	0.19	0.722
0.35	0.38	0.510	0.37	0.580
0.40	0.77	0.236	0.81	0.202
0.45	0.72	0.298	0.91	0.164
0.50	0.62	0.522	0.95	0.122
0.55	0.81	0.236	0.92	0.370
0.60	0.88	0.318	0.93	0.312
0.65	1.00	0.000	0.97	0.158
0.70	0.00	1.000	0.93	0.312
0.75	0.00	1.000	0.97	0.158
0.80	0.00	1.000	0.93	0.312

However, differences were detected in terms of the connectivity of each individual lipid in the two networks. The 10 most differentially connected lipids are listed in Table 2. From those, Cer(d18:1/16:0) (L20) and Cer(d18:1/24:1) (L25) have previously been related to an increased risk of CVD death. Two of the lipids in Table 2 are significant on 0.05 level: Cer(d18:1/16:0) (L20) and LacCer(d18:1/24:0) (L44). They are important hub lipids, binding two modules that are isolated in the control network into one larger module in the case network. The hub lipids are circled in Fig. 1 and 2.

**Table 2:** The 10 most differentially connected lipids for MI LURIC data based on the test for differential connectivity of individual lipids between case and control groups.

Lipid	Abbreviation	*d*	p-value
Cer(d18:1/16:0)	L20	0.078	0.014
LacCer(d18:1/24:0)	L44	0.075	0.038
Cer(d18:1/26:1)	L26	0.098	0.052
Cer(d18:1/24:1)	L25	0.072	0.074
Cer(d18:1/20:0)	L22	0.072	0.088
DAG 16:0/18:2	L28	0.090	0.090
Cer(d18:1/22:0)	L23	0.070	0.092
GlcCer(d18:1/20:0)	L36	0.074	0.096
SM (d18:1/16:1) (d18:1/15:2-OH)	L79	0.070	0.100
Cer(d18:1/18:0)	L21	0.068	0.108

For comparative purposes of the benefits of MI, it is impossible to do a proper complete case analysis as there are no patients with fully observed lipid profiles. Instead, we selected a subgroup of lipids that were observed at least from 90% of the patients and then selected the complete cases with these lipids fully observed. This resulted in 52 lipids and an effective sample size of 310. Based on the test of differential network structures with minimum module size *m* = 3 and connectivity threshold *ɛ* = 0.4, case and control groups did not differ significantly on 0.05 level (p = 0.202). The result is very similar to the one obtained by the MI approach. For any other moderate choice of *ɛ*, the differences in modular structures remain non-significant. The results indicate similar test results as in the MI approach as shown in Table 1. However, complete case analysis (not shown) was unable to identify clear modules among case and control networks as lipids seemed to form one large module despite increasing the threshold parameter *ɛ*. It is noteworthy, that we started with a fewer number of lipids for the complete case analysis compared to the number of lipids after imputation in the MI data. Also, the effective sample size is smaller and thus relationships between lipids are weaker. For example, in the complete case analysis, after choosing *ɛ* = 0.4, the case network consists of two modules including 35 lipids and control network of 37 lipids in one module. Thus, larger number of the lipids are one of the advantages of the MI approach. Threshold parameter *ɛ* = 0.5 resulted in the lowest p-value in the complete case analysis, but even then both networks consist of single modules with 26 lipids in the case network and 24 lipids in the control network.

In the complete case analysis, testing for the differential connectivity of a single lipid resulted in two lipids that were differentially connected between case and control groups, Gb3(d18:1/24:0) (L32) and LPC 16:0 (L40). LPC 16:0 (L40) did not come up in the previous analysis and it includes only two missing values in the original data set. LPC 16:0 (L40) is dropped out in the case group network, but in the control network it is connected to seven other lipids. However, all these connections are weak and the lipid is dropped out of the network, even when choosing a low value for threshold parameter *ɛ*. In the control group network, Gb3(d18:1/24:0) (L32) is connected only to one lipid and in the case group network to eight other lipids. Most of the other differentially connected top lipids in this analysis belonged to the ceramide or glucosylceramide classes. It is noteworthy, that most of the lipids that include left-censored values are left out of the analysis and thus the results may be biased.

The marginal analysis, using one lipid at a time individually and not in a network, for the MI data gave us 16 lipids that have significantly different mean concentrations between cases and controls. These lipids are listed in Tables 3 and 4. Marginal analysis found the two same risk related lipids, Cer(d18:1/16:0) (L20) and Cer(d18:1/24:1) (L25), that had differential connections in the case and control networks. LacCer(d18:1/24:0) (L44) is significantly differentially connected between case and control networks. Also Cer(d18:1/24:0) (L24) shares a similar structure with several other ceramides that came up in the networks. As a summary, marginal analysis mostly identified the same or related differing lipids as the differential network analysis. However, it is interesting to observe that none of the PC or PE lipids, that were differentially expressed in the marginal analysis, turned out to be differentially expressed in the network analysis. Majority of the lipids in the network analysis are from the ceramide class. Ceramides are responsible for mediating cell-stress responses and the regulation of cell death and cell ageing. In this particular experiment the differential nature of ceramides is quite relevant.

**Table 3:** The 13 imputed lipids having significantly different mean concentrations between case and control groups by the marginal analysis implemented by using Rubin’s rules.

Lipid	Abbreviation	$\hat{\beta }$	$\text{var}(\hat{\beta })$	*F*	p-value
PC 16:0/18:2	L50	-0.002	0.027	0.0002	0.011
SM (d18:1/18:0)	L81	0.006	0.043	0.0009	0.024
PC O-18:0/20:4-alkyl	L70	-0.007	0.055	0.0009	0.024
PE 18:0/20:4	L74	-0.010	0.061	0.0016	0.032
GlcCer(d18:1/18:0)	L35	-0.008	0.036	0.0018	0.034
DAG 16:0/18:1	L27	-0.012	0.059	0.0024	0.039
SM (d18:1/18:1)	L82	-0.010	0.043	0.0025	0.040
Gb3(d18:1/22:0)	L31	-0.012	0.046	0.0029	0.043
PC 18:0/18:1	L55	-0.011	0.041	0.0029	0.043
CE 19:2 oxCE 680.6	L11	-0.018	0.106	0.0031	0.044
SM (d18:1/14:0) (d18:1/13:1-OH)	L76	-0.011	0.035	0.0034	0.046
PC 16:0/18:1	L49	0.012	0.033	0.0035	0.047
LacCer(d18:1/22:0)	L43	0.013	0.042	0.0037	0.049

**Table 4:** The three fully observed lipids having significantly different mean concentrations between case and control groups by the marginal analysis.

Lipid	Abbreviation	$\hat{\beta }$	S.E.($\hat{\beta }$)	*t*	p-value
Cer(d18:1/16:0)	L20	0.110	0.032	3.469	0.0005
Cer(d18:1/24:0)	L24	-0.112	0.032	-3.484	0.0005
Cer(d18:1/24:1)	L25	0.061	0.031	1.982	0.0481

## Discussion

Differential network analysis provides a formal statistical methodology to examine differences in lipidomic network structures under two biological conditions and to recognize the important distinguishing lipids. It responds to the acknowledged need for efficient analytical tools in the fields of lipidomics [39]. Compared to a lipid specific marginal analysis, a network analysis provides a tool to consider all the lipids simultaneously. Marginal analysis may identify the key lipids affected within a specific group of patients, but differential network analysis takes this information further by examining all the lipids simultaneously and investigating how they act together. It also allows us to visualize inter-lipid connections and find groups of lipids, so called modules, that are closely connected.

The major contribution of this paper is to provide a recipe to perform differential network analysis on multiple imputed lipidomic data. We showed that this approach coincides well with our complete case analysis among frequently detected lipids, but reduces the possibility of bias and adds network information on less frequently detected lipids. With the proposed multiple imputation scheme followed by the customized differential network analysis one can take full advantage of the data in the presence of missing values.

The present data indicated significant network associations between different lipid species within the same lipid classes, but perhaps more importantly, also between different lipid species originating from different lipid classes. These lipid species may contain a highly regulated fatty acid component, the property which is significantly affecting many lipid species across lipid classes. On the other hand, in circulation, the majority of the lipids are carried in different lipoproteins (e.g. HDL or LDL) and therefore the role of HDLs may explain for example the observed association between cholesteryl esters and phosphatidylcholines. Cer(d18:1/16:0) (L20) has an important role in connecting different SL species together. SLs are structurally very diverse, and ceramides are the backbone of all SL class [40–42]. The structural diversity of ceramides is based either on their long chain sphingoid base [43] or fatty acid composition [44], leading to a large variety of ceramides distinguished by specific structural modifications [13].

The earlier lipidomics findings [7] indicate for the first time that alterations in SL metabolism leading to changes in the fatty acid chain length of ceramides are highly relevant to CV risk. Specific SLs and in particular ceramides with a distinct molecular structure, Cer18:1/16:0 (L20) and Cer18:1/18:0 (L21), are associated with CV risk while for instance Cer18:1/24:0 (L24) appears to be protective. Remarkably, the Cer18:1/16:0 to Cer18:1/24:0 ratio seems a better predictor of clinical outcome than traditional risk factors such as LDL cholesterol [7]. Importantly, in this study, tests for differentially connected individual lipids succeeded in identifying Cer18:1/16:0 as a key metabolite for increased CV outcome risk. This observation is consistent with earlier findings and demonstrates the usefulness of the differential network analysis with the complex lipidomic data. Regardless the field of research, the network analysis approach and the implementation routines designed for this case study may as well be extended to other types of molecular data, such as the microarray gene expression and protein expression data.

Given the high prevalence of CAD associated mortality, prevention of fatal and non-fatal myocardial infarctions in CAD patients is a clinical challenge. The average annual mortality rate is generally between 1–3% and the annual rate of non-fatal events is 1–2% among stable CAD patients. However, at individual patient level the outcome event risk may vary considerably and, therefore, risk estimation tools are needed for better care and treatment optimization. To this date serum total cholesterol and LDL cholesterol are considered to be the markers of arteriosclerosis and its clinical manifestations such as acute coronary events. However, it has been shown in previous studies that LDL cholesterol levels fail to recognize a substantial proportion of patients at high risk for coronary events [5, 7]. Thus, there is a need for understanding the roles of many other lipid species in arteriosclerosis beyond LDL cholesterol and HDL cholesterol. Tarasov et al. [7] performed a study on prospective clinical samples of CAD patients for evaluating the value of different molecular lipids separately to establish their causal relationship with CAD. However, the lipids act in consort. We are not aware of any study where all the lipids are considered together simultaneously in a network setup in order to identify the differences in two network structures. The current results in identifying Cer18:1/16:0 as a key metabolite for increased cardiovascular outcome risk may help us to develop improved risk assessment tools for physicians and help developing new drugs with better clinical outcome.

Differential network analysis is based on statistical tests such as differential connectivity of a lipid in the presence of other lipids in two networks. Finding precise calibration for multiple hypotheses correction in such tests becomes problematic with the network setup. All the existing multiple hypotheses correction procedures assume that the multiple tests are independent (or weak dependent) of each other. In the network setup that cannot be assumed. However, these tests perform well in simulation studies and real data analysis. Also the end result may depend on what association measure and threshold are being used to construct the network. Guided by simulation studies in Gill et al [23] and Pihur et al [22], we have used PLS based scores in this work.

The computation demand of the permutation tests using PLS scores for overall difference between the two networks and differences between individual lipids is substantial due to repeating the multiple imputation step for each permuted data set. We conducted the analyses using a high-performance distributed-memory cluster. The computing time for each of the 500 imputation-permutation steps takes about twenty minutes, which makes parallel computing highly useful in this context. Without the parallel computing the computation time for one permutation test would be several days, where as the parallel computing decreases the total time to less than one hour.

As an alternative to the PLS model to find the association measures for the network construction one could use penalized sparse regression models such as adaptive elastic net [45]. Adaptive elastic net tends to select strongly correlating groups of predicting variables in the model together, or on the contrary, leave them all out. We have constructed a network with adaptive elastic net followed by a differential network analysis with a permutation test which provided similar result as the PLS based connectivity scores.
